# Abnormal heart rate responses to exercise in non-severe COPD: relationship with pulmonary vascular volume and ventilatory efficiency

**DOI:** 10.1186/s12890-024-03003-y

**Published:** 2024-04-17

**Authors:** Minghui Shi, Shiwei Qumu, Siyuan Wang, Yaodie Peng, Lulu Yang, Ke Huang, Ruoxi He, Feng Dong, Hongtao Niu, Ting Yang, Chen Wang

**Affiliations:** 1 National Center for Respiratory Diseases, No. 2 East Yinghua Road, Chaoyang District, 100029 Beijing, China; 2 Institute of Respiratory Medicine, Chinese Academy of Medical Sciences, No. 2 East Yinghua Road, Chaoyang District, 100029 Beijing, China; 3 National Clinical Research Center for Respiratory Diseases, No. 2 East Yinghua Road, Chaoyang District, 100029 Beijing, China; 4 Department of Pulmonary and Critical Care Medicine, Center of Respiratory Medicine, China-Japan Friendship Hospital, No. 2 East Yinghua Road, Chaoyang District, 100029 Beijing, China; 5 Department of Rehabilitation Medicine, China-Japan Friendship Hospital, 100029 Beijing, China; 6 Department of Clinical Research and Data Management, Center of Respiratory Medicine, China-Japan Friendship Hospital, 100078 Beijing, China; 7 Capital Medical University, 100069 Beijing, China; 8 Peking University Health Science Center, 100871 Beijing, China; 9 Chinese Academy of Medical Sciences and Peking Union Medical College, No. 2 East Yinghua Road, Chaoyang District, 100730 Beijing, China; 10 Fangzhuang Community Health Service Center, Capital Medical University, 100078 Beijing, China

**Keywords:** Abnormal heart rate response, Cardiac autonomic dysfunction, Pulmonary vascular volume, Ventilation efficiency, Chronic obstructive pulmonary disease

## Abstract

**Background:**

Despite being a prognostic predictor, cardiac autonomic dysfunction (AD) has not been well investigated in chronic obstructive pulmonary disease (COPD). We aimed to characterise computed tomography (CT), spirometry, and cardiopulmonary exercise test (CPET) features of COPD patients with cardiac AD and the association of AD with CT-derived vascular and CPET-derived ventilatory efficiency metrics.

**Methods:**

This observational cohort study included stable, non-severe COPD patients. They underwent clinical evaluation, spirometry, CPET, and CT. Cardiac AD was determined based on abnormal heart rate responses to exercise, including chronotropic incompetence (CI) or delayed heart rate recovery (HRR) during CPET.

**Results:**

We included 49 patients with FEV1 of 1.2–5.0 L (51.1–129.7%), 24 (49%) had CI, and 15 (31%) had delayed HRR. According to multivariate analyses, CI was independently related to reduced vascular volume (VV; VV ≤ median; OR [95% CI], 7.26 [1.56–33.91]) and low ventilatory efficiency (nadir VE/VCO2 ≥ median; OR [95% CI], 10.67 [2.23–51.05]). Similar results were observed for delayed HRR (VV ≤ median; OR [95% CI], 11.46 [2.03–64.89], nadir VE/VCO2 ≥ median; OR [95% CI], 6.36 [1.18–34.42]).

**Conclusions:**

Cardiac AD is associated with impaired pulmonary vascular volume and ventilatory efficiency. This suggests that lung blood perfusion abnormalities may occur in these patients. Further confirmation is required in a large population-based cohort.

**Supplementary Information:**

The online version contains supplementary material available at 10.1186/s12890-024-03003-y.

## Introduction

Chronic obstructive pulmonary disease (COPD) is an inflammatory disease of the airways, alveoli, and microvasculature with systemic effects [1]. Cardiac autonomic dysfunction (AD) predicts long-term mortality, even after adjusting for coronary anatomy and left ventricular function [2, 3]. It is evaluated using a range of variables, among which abnormal heart rate (HR) responses to exercise are particularly attractive owing to their simplicity, availability, and clinical significance. Abnormal HR responses include an inability to use most of the HR reserve during exercise (chronotropic incompetence [CI]) or an inability to immediately slow the HR after exercise (abnormal HR recovery).

Previous studies of AD focused on cardiovascular diseases (CVDs), which worsen cardiac pump function [4–7]. AD is also highly prevalent in respiratory diseases [8, 9]. However, for COPD, which results in approximately 3 million global deaths annually [1], the factors related to cardiac AD remain unclear [10]. Thus, cardiac AD should be assessed and considered during COPD management [11]. HR responses during incremental exercise to evaluate cardiac autonomic function may be inappropriate in severe COPD patients. Because of ventilatory limitations, they are more likely to stop exercising before they reach their real maximal HR. However, underestimating cardiac function may not occur in mild-moderate COPD, wherein respiratory abnormalities are less pronounced.

Few research studies have explored how COPD-related mechanisms affect cardiac autonomic function [12]. Since enlarged lung volume is associated with impaired cardiac function [13, 14], most studies focused on only the negative effects of airflow obstruction and lung hyperinflation [15, 16]. However, studies have not assessed the impact of pulmonary circulation abnormalities on cardiac AD in COPD, despite the confirmed negative effects of pulmonary vascular loss on cardiac structure and functionRecently, non-invasive methods have provided valuable information reflective of pulmonary blood perfusion. For example, a pulmonary vessel tree segmented using computed tomography (CT) can directly assess pulmonary vascular volume (VV) [17, 18]. Reduced ventilatory efficiency (elevated physiological dead space) shown via a cardiopulmonary exercise test (CPET) reflects vascular pruning and capillary hypoperfusion in COPD [19, 20]. 

Therefore, this study aimed to characterise the pulmonary structure, lung function, and cardiopulmonary exercise response features in non-severe COPD complicated with cardiac AD and determine associations between cardiac AD and pulmonary VV and ventilatory efficiency parameters.

## Materials and methods

### Study participants

We prospectively recruited outpatients with clinically stable COPD diagnosed at the China-Japan Friendship Hospital according to the global initiative for chronic obstructive lung disease (GOLD) guidelines [1]. All participants underwent medical interviews, pulmonary function tests (PFTs), chest CT, and CPET. Inclusion criteria were as follows: post-bronchodilator ratio between forced expiratory volume in 1 s and forced vital capacity (FEV_1_/FVC) < 70%, FEV_1_ > 50%; achieving maximal effort during the CPET recommended by the ATS/ACCP [21]. Exclusion criteria were as follows: (pulmonary hypertension) PH; left ventricular (LV) dysfunction; ischaemic heart disease; atrial fibrillation; valvular heart disease; cardiomyopathy; taking medications affecting HR; previous rehabilitation programmes.

The recruitment period was between July 2022 and July 2023. The study was approved by the institutional review board at the China-Japan Friendship Hospital (2022-KY-141), and written informed consent was obtained from all participants.

### Procedures

#### PFT and CT acquisition

Initially, patients underwent assessments of demographic data, medical history, smoking exposure, and respiratory symptoms. PFTs and chest CTs were then performed. Spirometry, body plethysmography, and diffusing lung capacity for carbon monoxide (D_LCO_) measurements were performed using automated equipment (Jaeger Masterscreen, Germany). [22] All volumetric CT scan images were obtained at full inspiration in the supine position. CT images were reconstructed with a slice thickness of 1 mm and a 1 mm increment, with a resolution of 512 × 512 mm, using a 320-slice CT (Genesis, Canon Medical System, Japan). Next, quantitative CT image analysis was performed using the FACT-Digital LungTM software (DeXin, Xi’an, China). Each pulmonary vessel tree was segmented, generating a vascular volume relative to the entire lung. Emphysema was quantified on full inspiration using low-attenuation areas of the lung (<-950 Hounsfield units).

#### CPET

On the second day of assessments, patients were instructed not to take inhaled bronchodilators (β2 agonists and anticholinergics), consume alcohol or stimulant drinks, or engage in vigorous exercise for 24 h. Standardised CPETs were conducted using an electromagnetically braked cycle ergometer (Jaeger Masterscreen, Germany), following the manufacturer’s guidelines [23]. A continuous ramp protocol was applied. After 2 min of unloaded pedalling (rest phase-0 W), a 3-min warm-up phase (20 W) followed. The test phase included 20 W/2 min load increments. Patients were instructed to pedal with 60–65 rotations per minute. Patients’ effort was maximal if two or more of the following criteria emerged: predicted maximal HR is achieved; predicted maximal oxygen uptake and/or a plateau is observed; RER > 1.10.

Measurements included standard gas exchange, breathing pattern parameters, HR (12-lead electrocardiography), and O_2_ saturation (pulse oximetry, SpO_2_). V-slope and ventilatory equivalent methods were used to determine the anaerobic threshold (AT). [23] Peak VO_2_ and RER were reported as the average values determined over the last 30 s of the CPET. O_2_ pulse during the peak exercise was calculated as VO_2_/HR. The nadir ventilatory equivalent for carbon dioxide (VE/VCO_2_) was defined as the lowest 30-second average data point observed during a CPET. The nadir VE/VCO_2_ has been used as an indicator of ventilatory efficiency because of its prognostic value and clinical utility [19, 20]. 

#### Endpoint analysis

Abnormal HR responses are identified by both CI and abnormal heart rate recovery (HRR). Chronotropic response (CR) is defined as [(peak HR–rest HR) / (age-predicted maximal HR–rest HR)]. CI would be diagnosed if CR failed to reach 80%. Abnormal HRR is defined as an HR that declines ≤ 12 beats/min in the first minute after exercise. Age-predicted maximum HR was obtained using an established formula (220-age).

### Statistical analysis

The normality of all data was tested using the Shapiro–Wilk test. Continuous variables are presented as mean ± standard deviation (SD) or median and interquartile range. Unpaired Student’s t-tests or Mann–Whitney U tests were performed for between-group (GOLD 1 and 2; CI and no CI; normal and abnormal HRR) comparisons of continuous variables. Categorical variables are presented as proportions. The X^2^ test was used to compare the frequencies of the categorical variables. Multivariate logistic regression analyses were used to determine the odds ratios between selected PFT, CT, and CPET parameters (independent variables) and CI or abnormal HRR (dependent variables) (online supplement). For multivariate regression, a forward likelihood ratio model was used with stepwise entry and removal criteria of 0.05 and 0.10, respectively. Continuous variables (e.g., PFT, CT, and CPET parameters) were converted to categorical forms, with the cut-off point set at the median of all samples.

Statistical significance was set at *p* < 0.05. All statistical analyses were performed using the Statistical Package for the Social Sciences version 26 (IBM).

### Patient and public involvement

Patients and the public were not involved in the design or conduct of this study.

## Results

### Study population

Out of sixty-five recruited patients, forty-nine were finally included (Fig. **S1****)**. Included patients were predominantly men (79.6%) and had a mean (SD) age of 62 (9) years. 26 patients were of GOLD 1 with a mean (SD) FEV_1_ of 94.9% (14.5%), while 23 were of GOLD 2 with a mean (SD) FEV_1_ of 67.2% (9.0%). There were no between-group differences in age, sex, body mass index, number of smokers, or presence of diabetes or hypertension between GOLD 1 and GOLD 2. The CT-derived emphysema severity and VV were not different. During CPET, GOLD 2 had lower exercise capacity (VO_2peak_), breath reserve, and ventilatory efficiency than GOLD 1 **(**Table 1**)**. GOLD 1 subjects had a mean (SD) VO_2peak_ of 88.7% (18.8%), while GOLD 2 subjects had a mean (SD) VO_2peak_ of 80.5% (13.5%).

**Table 1:** Demographic, imaging, resting pulmonary function, and cardiopulmonary exercise test measurements in patients by GOLD grades

	All patients (*n* = 49)	GOLD 1 (*n* = 26)	GOLD 2 (*n* = 23)
Demographics			
Sex, female, n (%)	10 (20.4)	5 (19.2)	5 (21.7)
Age, years	62 ± 9	62 ± 10	63 ± 9
Weight, kg	66.0 (61.0–76.5)	69.0 (61.0–78.0)	65.1 (60.0–71.0)
BMI, kg/m^2^	24.0 (21.8–26.6)	24.1 (22.0–26.9)	23.9 (21.7–26.5)
History of smoking, n (%)	30 (61.2)	15 (57.7)	15 (65.2)
History of hypertension or diabetes, n (%)	16 (32.7)	8 (30.8)	8 (34.8)
mMRC	1 (0–1)	1 (0–1)	1 (0–1)
CAT	10 (5–15)	8 (4–14)	11 (6–15)
Hb	145 (137–158)	145 (137–157)	144 (138–158)
**Postbronchodilator pulmonary function**			
FEV_1_, %pred	81.9 ± 18.5	94.9 ± 14.5	67.2 ± 9.0*
FEV_1_/FVC, %	62.3 (57.4–66.9)	65.4 (61.7–68.0)	58.1 (49.7–62.6)*
**Prebronchodilator pulmonary function**			
D_LCO_, %pred	82.8 ± 20.9	82.1 ± 23.4	83.5 ± 18.1
IC, %pred	100.4 ± 20.3	108.4 ± 19.3	91.3 ± 17.8*
RV, %TLC	42.0 ± 7.3	38.7 ± 6.5	45.7 ± 6.5*
**Imaging**			
Lung volume, ml	5332.6 ± 1116.4	5597.4 ± 1202.9	5033.3 ± 947.9.8
LAA_950_, % the whole lung	9.6 (5.6–16.9)	9.9 (5.0–17.7)	9.6 (6.2–16.8)
VV, % the whole lung	3.6 ± 0.8	3.7 ± 0.8	3.4 ± 0.5
**Peak cardiopulmonary exercise test parameters (unless otherwise stated)**			
VO_2peak_, %pred	84.9 ± 16.9	88.7 ± 18.8	80.5 ± 13.5
VO_2peak_, ml/kg/min	21.8 ± 5.2	23.0 ± 6.2	20.5 ± 3.3*
VO_2_ at AT, %pred	50.9 ± 10.8	50.8 ± 9.4	51.1 ± 12.4
RER	1.2 ± 0.1	1.2 ± 0.1	1.2 ± 0.1
O_2_ pulse, %pred	92.3 ± 18.1	96.1 ± 21.9	88.1 ± 11.7
Rest HR, bpm	85 ± 13	86 ± 14	84 ± 12
Peak HR, bpm	146 ± 16	148 ± 17	144 ± 15
HRR at 1 min, bpm	129 ± 16	130 ± 18	128 ± 15
CR	0.86 ± 0.21	0.87 ± 0.23	0.84 ± 0.20
CI, n (%)	24 (49.0%)	12 (46.2%)	12 (52.2%)
Delayed HRR, n (%)	15 (30.6%)	5 (19.2%)	10 (43.5%)
BR, %	12.8 (7.2–31.0)	27.7 (12.8–36.8)	6.9 (0.4–10.7)*
Nadir VE/VCO_2_	29.5 (27.8–32.2)	29.4 (26.5–32.6)	30.0 (28.1–31.6)

*Abbreviations* BMI = body mass index; mMRC = modified Medical Research Council dyspnea scale, CAT = chronic obstructive pulmonary disease assessment test; FEV_1_ = forced expiratory volume in 1 s; FEV_1_/FVC = ratio between FEV_1_ and forced vital capacity; D_LCO_=diffusing capacity of the lung for carbon monoxide; IC = inspiratory capacity; RV = residual volume; TLC = total lung capacity; LAA_950_ = low-attenuation areas of the lung below − 950 Hounsfield units; VV = vascular volume; VO_2_ = oxygen consumption; AT = anaerobic threshold; RER = respiratory exchange ratio; HR = heart rate; CR = chronotropic response; CI = chronotropic incompetence; HRR = heart rate recovery; BR = breath reserve; VE/VCO_2_ = ventilatory equivalent for carbon dioxide

Continuous data are presented as mean ± standard deviation or median, interquartile range. Categorical data are presented as n (%)

*P < 0.05 vs. GOLD 1

### Demographic, PFT, CT, CPET parameters, and CI

Table 2 describes demographic, lung function, lung imaging, and cardiopulmonary exercise response variable differences among patients with and without CI. Participants who developed CI had a lower resting D_LCO_, greater lung hyperinflation, and blood VV abnormalities. Regarding CPET parameters, in addition to a significantly lower VO_2peak_ and VO_2_ at AT, those with CI had significantly higher nadir VE/VCO_2_, indicating reduced ventilatory efficiency.

**Table 2:** Demographic, imaging, resting pulmonary function and cardiopulmonary exercise test measurements in patients with or without CI

	No CI (*n* = 25)	CI (*n* = 24)	Effect size (95% CI)	P-value
Demographics				
Sex, female, n (%)	7 (28.0)	3 (12.5)		0.289
Age, years	63 ± 10	62 ± 9		0.436
Weight, kg	66.0 (60.0–76.5)	66.1 (62.2–77.0)		0.689
BMI, kg/m^2^	24.3 (21.9–27.0)	23.7 (21.6–26.3)		0.575
History of smoking, n (%)	14 (56.0)	16 (66.7)		0.444
History of hypertension or diabetes, n (%)	10 (40.0)	6 (25.0)		0.263
mMRC	1 (0–1)	1 (0–1)		0.530
CAT	10 (6–13)	11 (5–16)		0.679
**Postbronchodilator pulmonary function**				
FEV_1_, %pred	83.1 ± 16.1	80.7 ± 20.9		0.655
GOLD grade, GOLD 1, n (%)	14 (56.0%)	12 (50.0%)		0.674
FEV_1_/FVC, %	62.8 (58.3–67.0)	61.1 (54.6–66.8)		0.484
**Prebronchodilator pulmonary function**				
D_LCO_, %pred	90.9 ± 18.1	74.4 ± 20.7	16.5 (5.3–27.6)	0.005
IC, %pred	105.8 ± 20.3	94.7 ± 19.1	11.0 (-0.3–22.4)	0.056
RV, %TLC	42.8 ± 6.4	41.2 ± 8.2		0.464
**Imaging**				
Lung volume, ml	5168.7 ± 1163.2	5503.4 ± 1062.8		0.299
LAA_950_, % the whole lung	8.4 (5.0–14.6)	11.2 (6.6–20.6)		0.368
VV, % the whole lung	3.7 ± 0.8	3.4 ± 0.7	0.40 (-0.03–0.82)	0.07
**Peak cardiopulmonary exercise test parameters (unless otherwise stated)**				
VO_2peak_, %pred	76.3 ± 13.6	93.2 ± 15.7	16.9 (8.5–25.4)	< 0.001
VO_2_ at AT, %pred	47.2 ± 11.5	54.5 ± 8.8	7.3 (1.4–13.2)	0.016
RER	1.2 ± 0.1	1.2 ± 0.1		0.224
O_2_ pulse, %pred	92.3 ± 18.1	93.3 ± 19.0		0.706
Rest HR, bpm	85 ± 13	87 ± 14		0.438
Peak HR, bpm	146 ± 16	157 ± 13	21.7 (15.0–28.3)	< 0.001
HRR at 1 min, bpm	129 ± 16	137 ± 15	16.4 (8.3–24.5)	< 0.001
CR	1.02 ± 0.17	0.69 ± 0.07	0.33 (0.25–0.41)	< 0.001
BR, %	12.8 (7.2–31.0)	12.6 (7.6–27.7)		0.889
Nadir VE/VCO_2_	28.4 (27.3–30.8)	31.5 (28.3–36.0)	-4.0 (-6.9– -1.1)	0.014

*Abbreviations* CI = Chronotropic Incompetence; BMI = body mass index; mMRC = modified Medical Research Council dyspnoea scale, CAT = chronic obstructive pulmonary disease assessment test; FEV_1_ = forced expiratory volume in 1 s; FEV_1_/FVC = ratio between FEV_1_ and forced vital capacity; D_LCO_=diffusing capacity of the lung for carbon monoxide; IC = inspiratory capacity; RV = residual volume; TLC = total lung capacity; LAA_950_ = low-attenuation areas of the lung below − 950 Hounsfield units; VV = vascular volume; VO_2_ = oxygen consumption; AT = anaerobic threshold; RER = respiratory exchange ratio; HR = heart rate; BR = breath reserve; VE/VCO_2_ = ventilatory equivalent for carbon dioxide

Continuous data are presented as mean ± standard deviation or median, interquartile range. Categorical data are presented as n (%)

**Fig. 1:**
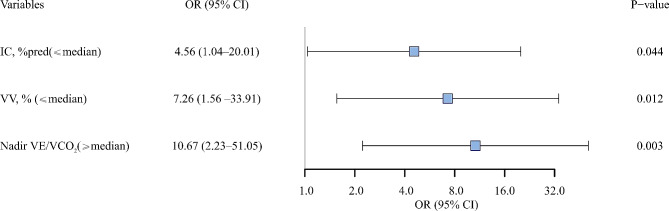
The relationship between PFT, CT, CPET parameters and CI. Adjusted OR (with upper and lower 95% CIs) for likelihood of having lung function, imaging and/or cardiopulmonary exercise response impairment in participants with CI compared with those without CI. *Abbreviations* PFT = pulmonary function test; CT = computed tomography; CPET = cardiopulmonary exercise test; CI = chronotropic incompetence; OR = odds ratio; CI = confidence interval; IC = inspiratory capacity; VV = vascular volume; VE/VCO_2_ = ventilatory equivalent for carbon dioxide

A multivariate logistic regression analysis included variables that exhibited a significant difference in univariate analysis or were consistently related to automatic cardiac function in previous literature [2, 3, 6, 7, 11, 15], with testing for multicollinearity (online supplement). Only IC, VV, and nadir VE/VCO_2_ independently predicted CI after adjustments (Fig. 1).

### Demographic, PFT, CT, CPET parameters, and abnormal HRR

Table 3 shows differences between patients with normal and abnormal HRR. Those with delayed HRR had an older age, more severe airflow limitations, gas exchange abnormalities, reduced lung vessel beds, and greater lung emphysema. During CPET, in addition to significantly lower VO_2peak_ and VO_2_ at AT, patients with delayed HRR had statistically significant lower ventilatory efficiency and breath reserve.

**Table 3:** Demographic, imaging, resting pulmonary function and cardiopulmonary exercise test measurements in patients with normal or delayed HRR

	Normal HRR (*n* = 34)	Delayed HRR (*n* = 15)	Effect size (95% CI)		P-value
Demographics					
Sex, female, n (%)	7 (20.6)	3 (20.0)			0.642
Age, years	60 ± 9	67 ± 9	-7.3 (-12.7– -1.8)		0.014
Weight, kg	69.5 (62.3–79.3)	63.9 (60.0–69.0)			0.059
BMI, kg/m^2^	24.4 (21.9–26.9)	22.9 (21.0–25.0)			0.193
History of smoking, n (%)	20 (58.8)	10 (66.7)			0.604
History of hypertension or diabetes, n (%)	14 (41.2)	2 (13.3)			0.097
mMRC	1 (0–1)	1 (0–1)			0.443
CAT	8 (3–13)	12 (8–16)			0.054
**Postbronchodilator pulmonary function**					
FEV_1_, %pred	84.5 ± 13.1	76.0 ± 26.7			0.138
GOLD grade, GOLD 1, n (%)	21 (61.8%)	5 (33.3%)			0.066
FEV_1_/FVC, %	64.6 (61.0–67.6)	53.5 (48.2–61.1)	9.6 (4.3–14.9)		0.003
**Prebronchodilator pulmonary function**					
D_LCO_, %pred	88.3 ± 20.8	70.4 ± 15.6	17.9 (5.8–29.9)		0.005
IC, %pred	102.2 ± 15.2	96.2 ± 29.0			0.348
RV, %TLC	41.1 ± 5.9	44.1 ± 9.8			0.183
**Imaging**					
Lung volume, mL	5267.6 ± 1128.7	5480.0 ± 1112.1			0.545
LAA_950_, % the whole lung	8.4 (4.2–13.1)	16.8 (9.6–23.6)	-7.1 (-12.0– -2.1)		0.008
VV, % the whole lung	3.8 ± 0.7	3.1 ± 0.7	0.7 (0.2–1.1)		0.005
**Peak cardiopulmonary exercise test parameters (unless otherwise stated)**					
VO_2peak_, % pred	88.9 ± 17.3	75.7 ± 12.0	13.2 (3.3–23.1)		0.005
VO_2_ at AT, % pred	52.2 ± 11.2	48.1 ± 9.4			0.219
RER	1.2 ± 0.1	1.2 ± 0.1			0.504
O_2_ pulse, % pred	94.1 ± 19.9	88.3 ± 13.1			0.301
Rest HR, bpm	85 ± 13	85 ± 13			0.933
Peak HR, bpm	150 ± 16	138 ± 14	12.0 (2.6–21.3)		0.011
HRR at 1 min, bpm	128 ± 17	131 ± 15			0.573
BR, %	22.3 (7.8–33.9)	9.5 (-30.2 to 16.1)	27.1 (9.2–45.0)		0.02
CR	0.89 ± 0.22	0.79 ± 0.19			0.169
Nadir VE/VCO_2_	28.4 (26.1–31.2)	31.6 (29.4–39.2)	-5.2 (-9.0– -1.3)		0.003

*Abbreviations* HRR = heart rate recovery; BMI = body mass index; mMRC = modified Medical Research Council dyspnoea scale, CAT = chronic obstructive pulmonary disease assessment test; FEV_1_ = forced expiratory volume in 1 s; FEV_1_/FVC = ratio between FEV_1_ and forced vital capacity; D_LCO_=diffusing capacity of the lung for carbon monoxide; IC = inspiratory capacity; RV = residual volume; TLC = total lung capacity; LAA_950_ = low-attenuation areas of the lung below − 950 Hounsfield units; VV = vascular volume; VO_2_ = oxygen consumption; AT = anaerobic threshold; RER = respiratory exchange ratio; HR = heart rate; BR = breath reserve; VE/VCO_2_ = ventilatory equivalent for carbon dioxide

Continuous data are presented as mean ± standard deviation or median, interquartile range. Categorical data are presented as n (%)

**Fig. 2:**
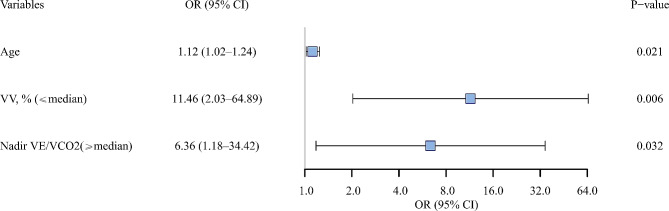
The relationship between PFT, CT, CPET parameters and delayed HRR. Adjusted OR (with upper and lower 95% CIs) for likelihood of having lung function, imaging and/or cardiopulmonary exercise response impairment in participants with delayed HRR compared with those with normal HRR. *Abbreviations* PFT = pulmonary function test; CT = computed tomography; CPET = cardiopulmonary exercise test; HRR = heart rate recovery; OR = odds ratio; CI = confidence interval; VV = vascular volume; VE/VCO_2_ = ventilatory equivalent for carbon dioxide;

The logistic regression model revealed lung vessel volume and nadir VE/VCO_2_ as independent predictors for delayed HRR (Fig. 2).

## Discussion

Our main findings were as follows: (1) in COPD, cardiac AD identified by CI and delayed HRR was independently associated with CT indices of pulmonary VV abnormalities; (2) cardiac AD was independently related to high nadir VE/VCO_2_ during the CPET.

After adjusting for relevant factors in our study, those with CI or delayed HRR had significantly reduced CT-derived VV, a valid index of small vessel loss [17, 18]. A high nadir VE/VCO_2_ ratio, which indicates increased VD/VT during exercise, was also confirmed as an independent predictor of abnormal HR responses [19, 20, 24]. Thus, our results suggest that patients with cardiac AD are more likely to have reduced pulmonary blood perfusion, which is theoretically in line with the negative effects of pulmonary vascular loss on cardiac structure and function. It is routinely assumed that AD is secondary to chronic sympathetic system overactivation. Pulmonary vascular remodeling could lead to higher left ventricular (LV) mass and LV diastolic dysfunction, then increase sympathetic system overactivation [16, 25–27]. 

Our study identifies the susceptibility to cardiac AD of one specific subset of patients, the so-called “pulmonary vascular phenotype”. In most COPD patients, loss of the pulmonary vascular bed can be attributed to distal airway damage and alveolar destruction. However, in some patients with mild-to-moderate COPD, vascular pruning and capillary hypoperfusion can exist in both emphysematous and non-emphysematous lung regions, especially in those who are ever-smokers [28–32]. In this subset of individuals, endothelial injury and pulmonary vascular remodelling may occur before developing airflow obstruction or emphysema, the so-called “pulmonary vascular phenotype” [33]. Despite poorer prognosis due to early pulmonary vascular damage [34], this phenotype has been overlooked in most researchand clinical practice. By supplementing cardiac performance for this subset, our finding have stressed that they may be exposed to a higher risk of cardiac dysfunction, appealing more clinical attention and directed research. In addition, pulmonary circulation abnormalities in COPD require better screening tools, with previous studies only focusing on patients with severe pulmonary vascular disease. However, a much larger group with mild disease only exhibited a steep increase in pulmonary arterial pressure during exercise [33, 35]. Based on our findings, abnormal HR responses during exercise tests (e.g., field walking tests and CPET) have the potential to be a non-invasive, accessible, effective screening tool for pulmonary circulation abnormalities. The therapy of the pulmonary vascular phenotype of COPD is challenging. Although optimized treatment of the underlying lung disease is suggested, there are no data to show that this optimized treatment improves PH. On the basis of sentinel studies, long-term oxygen therapy may reduce the progression of PH. As for PAH drugs, we need prospective studies providing evidence that these patients do profit from such therapy before it can be recommended [33]. 

Cardiac AD exists regardless of the severity of COPD and has predictive value for quality of life and mortality; we suggest physicians pay attention to it during clinical management [36–38]. These patients may not experience sufficient symptom relief from typical COPD treatment. Thus, prospective studies should determine whether patients benefit from rehabilitative interventions, such as exercise training, which have been shown to be effective in those with CVDs [33, 35, 39]. 

The relationship between AD and FEV_1_ remains controversial. For example, Chicks demonstrated that delayed HRR was independent of FEV_1_, whereas Hulo reported a higher prevalence of CI as FEV_1_ decreased [40, 41]. In addition, Cherneva et al. reported a relationship between cardiac AD and lung hyperinflation [16]. Our results showed that these variables could not separate the two groups in multivariate analysis. However, we also observed that delayed HRR was independently associated with a reduced FEF_50–75%_, which is also closely related to airflow limitation. The controversies regarding the association between AD and different pulmonary function parameters are probably due to different study designs and protocol performances that should be determined in a larger population-based sample.

### Limitations

This study had limitations. First, a selection bias may have occurred because our analysis only included mild-moderate patients who were able to achieve their maximal exercise capacity; however, HR responses during or after an incremental maximal exercise test would underestimate the cardiac function of COPD patients who stop tests earlier because of severe ventilatory limitation. HR variability may be a more precise measure of cardiac AD in severe COPD. Nonetheless, for mild-moderate COPD, the literature supports using HR responses to exercise as the biomarker of autonomic function to easily predict clinical outcomes in the clinical setting. Second, the relatively small sample size and multiple tests may have led to false commission or the omission of results (type I/II error). Larger population-based cohorts are needed to confirm the conclusion. Additionally, it would be more precise to include dynamic inspiratory capacity (ICdyn) as an adjusted factor when exploring the correlation between low pulmonary vascular volume and cardiac AD. It is our limitation not to serially measure ICdyn. However, several measures were taken to make our findings more robust: static hyperinflation was assessed by IC at rest and quantified emphysema, which correlates closely to DH; both emphysema and IC were included in the multivariate logistic regression model. Finally, with limited resources, we were unable to evaluate brain natriuretic peptide level and echocardiography for each patient. However, considering that PH and LV dysfunction affect cardiac automatic function, we did taken many other measures to exclude these patients, such as asking medical history, collecting medical records and performing chest CT. To confirm our results, we would also carry out research including essential accessory examinations in the future.

## Conclusion

We are the first to report that cardiac AD is linked to reduced pulmonary VV and low ventilatory efficiency in COPD based on chest CT, spirometry, and CPET characterization. Impaired VV and ventilatory efficiency pointed to reduced pulmonary blood perfusion, possibly affecting cardiac fulfilment.

### Electronic supplementary material

Below is the link to the electronic supplementary material.

Supplementary Material 1

## Data Availability

The datasets generated and/or analyzed during the current study are not publicly available due to patients’ personal information but are available from the corresponding author upon reasonable request.
