# Supplementary material for: Abnormal heart rate responses to exercise in non-severe COPD: relationship with pulmonary vascular volume and ventilatory efficiency
Source: BMC Pulm Med. 2024 Apr 17;24:183. doi: 10.1186/s12890-024-03003-y (PMC11022473; doi:10.1186/s12890-024-03003-y)
Supplement: Supplementary file 1 — Supplementary Material 1 [file 12890_2024_3003_MOESM1_ESM.docx]

**Abnormal heart rate responses to exercise in non-severe COPD: Relationship with pulmonary vascular volume and ventilatory efficiency**

***Materials and Methods***

*Statistical analysis covariates*

When performing the multivariate logistic regression, we considered variables that exhibit potential relations to chronotropic incompetence(CI) in univariate analysis such as diffusing capacity of the lung for carbon monoxide(D_LCO_), vascular volume(VV), and nadir ventilatory equivalent for carbon dioxide(VE/VCO_2_). We also considered variables being confirmed consistently related to cardiac autonomic function or COPD severity in previous literature such as age, smoking history, history of systemic hypertension and/or diabetes, severity of airflow limitation[forced expiratory volume in 1 second(FEV_1_)], inspiratory capacity (IC), pulmonary emphysema burden [low-attenuation areas of the lung below -950 Hounsfield units(LAA_950_)] and breath reserve(BR) [1-6]. Thus, after testing multicollinearity, for CI, the final model included age, smoking history, history of systemic hypertension and/or diabetes, FEV_1_, D_LCO_, IC, VV, LAA_950_, nadir VE/VCO_2_ and BR.

Similar procedures were followed when performing the multivariate regression analysis for abnormal heart rate recovery(HRR). For HRR, the final model included age, smoking history, history of systemic hypertension and/or diabetes, FEV_1_, D_LCO_, IC, VV, LAA_950_, nadir VE/VCO_2_ and BR.

**References**

1. Lauer MS, Pashkow FJ, Larson MG, Levy D. Association of cigarette smoking with chronotropic incompetence and prognosis in the Framingham Heart Study. Circulation. 1997;96(3):897-903.

2. Mohammed J, Meeus M, Derom E, Da Silva H, Calders P. Evidence for Autonomic Function and Its Influencing Factors in Subjects With COPD: A Systematic Review. Respir Care. 2015;60(12):1841-51.

3. Andreas S, Anker SD, Scanlon PD, Somers VK. Neurohumoral activation as a link to systemic manifestations of chronic lung disease. Chest. 2005;128(5):3618-24.

4. Mayr AK, Wieser V, Funk GC, Asadi S, Sperk I, Urban MH, Valipour A. Impaired Spontaneous Baroreceptor Reflex Sensitivity in Patients With COPD Compared to Healthy Controls: The Role of Lung Hyperinflation. Front Med (Lausanne). 2021;8:791410.

5. Cherneva RV, Youroukova VM, Cherneva ZV. Dynamic hyperinflation, chronotropic intolerance and abnormal heart rate recovery in non-severe chronic obstructive pulmonary disease patients-reflections in the mirror. Pulmonology 2022;28(6):440-448.

6. Zweerink A, van der Lingen ACJ, Handoko ML, van Rossum AC, Allaart CP. Chronotropic Incompetence in Chronic Heart Failure. Circ Heart Fail. 2018;11(8):e004969.

**Supplement Tables and Figures**

**Table S1.** Multivariate logistic regression analysis between PFT, CT, CPET parameters and abnormal HRR.

|  | **OR** | **95% CI** | **P-value** |
| --- | --- | --- | --- |
| Age | 1.12 | 1.02–1.24 | 0.021 |
| VV, % (≤median) | 11.46 | 2.03–64.89 | 0.006 |
| Nadir VE/VCO_2_ (≥median) | 6.36 | 1.18–34.42 | 0.032 |

Variables included in the model: age, smoking history, history of systemic hypertension and/or diabetes, FEV_1_, D_LCO_, IC, VV, LAA_950_, nadir VE/VCO_2_ and BR.

Abbreviations*:* HRR= heart rate recovery; PFT= pulmonary function test; CT= computed tomography; CPET= cardiopulmonary exercise test; OR= odds ratio; CI= confidence interval; FEV_1_= forced expiratory volume in 1 second; D_LCO_=diffusing capacity of the lung for carbon monoxide; IC = inspiratory capacity; LAA_950_= low-attenuation areas of the lung below -950 Hounsfield units; VV= vascular volume; BR= breath reserve; VE/VCO_2_= ventilatory equivalent for carbon dioxide

**Table S2.** Multivariate logistic regression analysis between PFT, CT, CPET parameters, and CI

|  | **OR** | **95% CI** | **P-value** |
| --- | --- | --- | --- |
| IC, %pred (≤ median) | 4.56 | 1.04–20.01 | 0.044 |
| VV, % (≤ median) | 7.26 | 1.56 –33.91 | 0.012 |
| Nadir VE/VCO_2_ (≥ median) | 10.67 | 2.23–51.05 | 0.003 |

Variables included in the model: age, smoking history, history of systemic hypertension and/or diabetes, FEV_1_, D_LCO_, IC, VV, LAA_950_, nadir VE/VCO_2_ and BR.

Abbreviations*:* CI= chronotropic incompetence; PFT= pulmonary function test; CT= computed tomography; CPET= cardiopulmonary exercise test; OR= odds ratio; CI= confidence interval; FEV_1_= forced expiratory volume in 1 second; D_LCO_=diffusing capacity of the lung for carbon monoxide; IC = inspiratory capacity; LAA_950_= low-attenuation areas of the lung below -950 Hounsfield units; VV= vascular volume; BR= breath reserve; VE/VCO_2_= ventilatory equivalent for carbon dioxide

Fig. S1. Study flowchart.


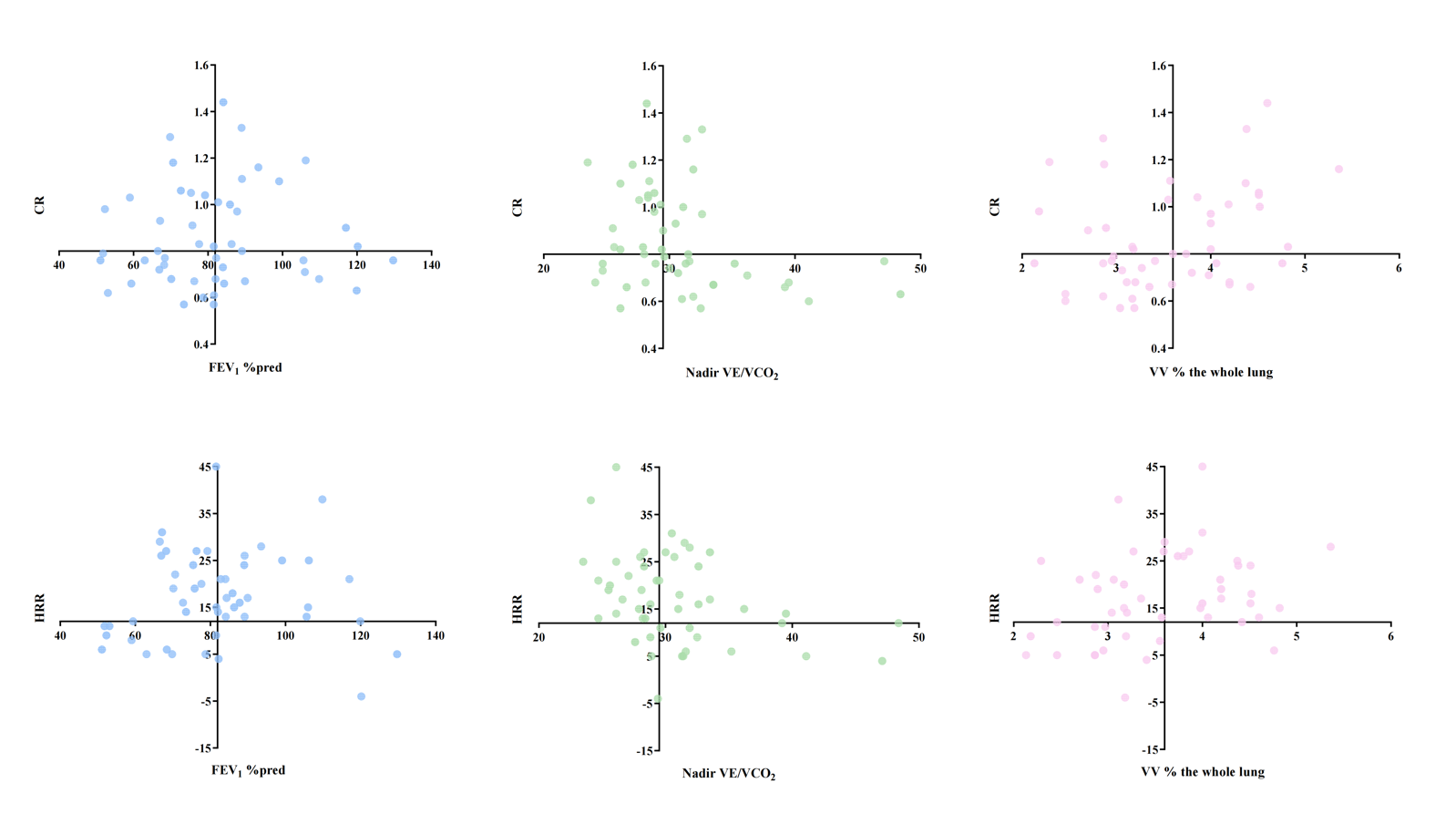


Fig. S2. The relationship between FEV_1_, nadir VE/VCO2, vascular volume and cardiac AD.

Abbreviations*:* CI= chronotropic incompetence; HRR= heart rate recovery; FEV_1_= forced expiratory volume in 1 second; VV= vascular volume; VE/VCO_2_= ventilatory equivalent for carbon dioxide;
